# Genome-wide association studies identified novel SNPs associated with efficient biological nitrogen fixation in chickpea (*Cicer arietinum* L.)

**DOI:** 10.3389/fpls.2025.1652315

**Published:** 2025-11-12

**Authors:** Chandana B. S, Rohit Kumar Mahto, Rajesh Kumar Singh, Ramachandra V, K. K. Singh, Sunita Kushwah, Gera Roopa Lavanya, Himabindu Kudapa, Vinod Kumar Valluri, Anil Kumar Vemula, Raju Ratan Yadav, Lal Bahadur Yadav, H. D. Upadhyaya, Aladdin Hamwieh, Rajendra Kumar

**Affiliations:** 1 Division of Genetics, ICAR-Indian Agricultural Research Institute, New Delhi, India; 2 School of Biotechnology, Institute of Science, BHU, Varanasi, India; 3 Department of Genetics and Plant Breeding, UAS, GKVK, Bengaluru, India; 4 ICAR-Indian Agricultural Research Institute (IARI) Regional Station, Pusa, Bihar, India; 5 Krishi Vigyan Kendra (KVK), Vaishali (Dr. Rajendra Prasad Central Agriculture University- Pusa), Bihar, Samastipur, India; 6 Department of Genetics and Plant Breeding, SHUATS, Naini, Prayagraj, India; 7 International Crops Research Institute for the Semi-Arid Tropics (ICRISAT), Patancheru, Telangana, India; 8 Department of Molecular Biology and Genetic Engineering, Govind Ballabh Pant University of Agriculture and Technology, Pantnagar, Uttarakhand, India; 9 Department of Plant Pathology, Govind Ballabh Pant University of Agriculture and Technology, Pantnagar, Uttarakhand, India; 10 Plant Genome Mapping Laboratory, University of Georgia, Athens, GA, United States; 11 International Center for Agricultural Research in the Dry Areas (ICARDA), Giza, Egypt

**Keywords:** chickpea, GWAS, KASP, MTAs, root nodulation

## Abstract

Chickpea (*Cicer arietinum* L.) is the second most important food legume crop, capable of converting atmospheric nitrogen (N_2_) into ammonia (NH_3_) in symbiotic association with *Mesorhizobium cicero* through a process called biological nitrogen fixation (BNF). BNF shows promise in effectively diminishing reliance on exogenous nitrogen applications, enhancing soil sustainability and productivity in pulse crops. Notably, there are limited studies on the molecular basis of root nodulation in chickpea. In order to identify new sources of highly nodulating genotypes and gain deep insights into genomic regions governing BNF, a diverse chickpea global germplasm collection (284) was evaluated for nodulation and yield traits in four different environments in an augmented randomized block design. The genotypes exhibited significant trait variation, encompassing all traits under study. Correlation analysis revealed a significant positive correlation of nodulation traits on yield within the chickpea population. The genotypes ICC 7390, ICC 15, ICC 8348, and ICC 2474 were identified as high nodulating across the locations. Genome-wide association studies (GWAS) identified noteworthy and stable marker–trait associations (MTAs) linked to the traits of interest. For the traits number of nodules (NON) and nodule fresh weight (NFW), 65 and 109 significant MTAs were identified, respectively. In addition, two SNPs, Ca1pos289.52482.1 and 6_33340878, identified in our earlier studies were validated by independent population studies, which are crucial in evaluating the accuracy and reliability of the projections. Subsequent analysis revealed that a substantial proportion of these MTAs were situated within intergenic regions, with the potential to modulate genes associated with the focal traits. The candidate genes identified could be converted to Kompetitive allele-specific PCR (KASP) markers and exploited in marker-assisted breeding, accentuating their impact on future chickpea breeding efforts.

## Introduction

The globally grown third most important pulse chickpea (*Cicer arietinum* L.), a self-pollinated diploid crop having a 2n=2x=16 chromosome number, is mainly grown during the winter season as an annual crop. The crop is cultivated over an area of 14.84 million ha with an average yield of 1.0 t/ha and a total production of 15.08 million tons (FAOSTAT, 2023). India is the leading producer of chickpeas, with an annual production of 11.5 million tons (Grasso et al., 2022). The chickpea seed matrix is composed of carbohydrates (50%–58%), protein (15%–22%), moisture (7%–8%), fat (3.8%–10.20%), and micronutrients (<1%) (Milán-Carrillo et al., 2007; Misra et al., 2016; Katoch et al., 2016; Kumar et al., 2021; Mittal et al., 2023). Moreover, chickpea is a rich source of minerals including iron (Fe), zinc (Zn), and selenium (Se) (Sharma et al., 2013).

Chickpea, like other fellow legumes, has the ability to fix nitrogen through symbiotic association. Symbiotic nitrogen fixation (SNF), a form of biological nitrogen fixation (BNF), is an important biological event that provides 97% of a plant’s total N requirement and agronomical and environmental benefits, allowing legumes to grow efficiently under nitrogen-limiting conditions (Peoples and Craswell, 1992). Because of the unique ability of the host plant to form a symbiotic relationship with a group of nitrogen-fixing bacteria called rhizobia, legumes represent an important and diverse group of plants, harboring 50%–70% of BNF, leading to a terrestrial input of 40–50 million tons of nitrogen per year (Vitousek et al., 1997). Chickpea carries out SNF by forming a symbiotic interaction with *Mesorhizobium ciceri* (Greenlon et al., 2019). This process of symbiosis and nodulation leading to N_2_ fixation is quite complex and tightly regulated, but very scantily understood at the molecular level. Some significant microbiological work on chickpea root nodule symbiosis with a focus on phenotypic and genotypic diversity among symbiotic vs. non-symbiotic bacteria revealed that 55% of isolated bacteria belong to the *Mesorhizobium* genus (Benjelloun et al., 2019). Recently, experiments were conducted on root nodulation-specific chickpea genotypes in varied soils (Chandana et al., 2023; Plett et al., 2021). The *Mesorhizobium* strains such as LMG15046, CC1192, XAP4, XAP10, and LMG14989 were highly effective strains for nodulation and growth promotion in chickpea (Wanjofu et al., 2022). Frailey et al. (2022) reported two mutations of rn1 and rn4 in the genotypes of PM233 and PM 405 that result in the absence of nodulation. The BNF is necessary for an environmentally friendly and sustainable agricultural production.

Understanding genes and genomic regions that support BNF is essential to increase efficiency and utilize the benefits. There have been few large-scale studies comparing chickpea nodulation and nitrogen production across multiple genotypes. Recent advancements in next-generation sequencing technologies as well as the availability of applicable tools such as genome-wide association studies (GWAS) have aided in the identification of single-nucleotide polymorphisms (SNPs) for nutritional and agronomic traits (Roorkiwal et al., 2022; Istanbuli et al., 2024). The release of whole-genome sequencing (WGS) and whole-genome resequencing (WGRS) data of 3,366 chickpeas (Varshney et al., 2013, 2021) can facilitate the identification of genomic regions for traits associated with nodulation through genome-wide association mapping. In this regard, a newly constructed association panel for root nodulation traits is required by characterizing them through extensive phenotyping and genotyping. Understanding nodulation and N-fixation in chickpea is important to maximize the benefit of N-fixation and reduce reliance on nitrogenous fertilizers. With this background, we have carried out the current research on GWAS for root nodulation traits in chickpea as presented further.

## Materials and methods

### Plant material and experimental procedure

An association panel consisting of a collection of 284 diverse germplasms obtained and extracted from ICRISAT (Upadhyaya, 2003) along with four checks, BG 372, BG 3022, BG 547, and BG 1105, were used for GWAS analysis on nodulation traits. The experimental trials for the association panel were conducted at four environmental locations, namely, IARI, New Delhi (location 1; 28°38'24.0252” N latitude, 77°10'26.328*”* E longitude and 228.6 m AMSL) having sandy clay loam soils; Sam Higginbottom University of Agriculture, Technology and Sciences (SHUATS), Naini, Prayagraj (location 2; 25°24'41.27” N latitude, 81°51'3.42” E longitude and 98 m AMSL) with clay loam to sandy loam soil; KVK, Vaishali, Dr. Rajendra Prasad Central Agricultural University (RPCAU), Samastipur (location 3; 25°86'29.679”N latitude, “85°78'10.263”E longitude to accurately reflect the geographical location, and 52 m AMSL) with sandy loam soil; and IARI Regional station, Pusa, Bihar (location 4; 25°54'56.16” latitude, 85°40'24.9564*”* longitude, and 52 m AMSL) with alluvial soils during the year 2020–2021. Experimental trials consisted of 284 germplasm lines. The field in all the locations was divided into four blocks with four checks in each block. Each check was replicated three times in all the blocks. The experimental design was an augmented random block design with a row length of 5 m, a row spacing of 60 cm, and a plant-to-plant distance of 10 cm (**Supplementary Table 1**). Observations were taken for the following six traits associated with nodulation: number of nodules per plant (NON/plant), nodule fresh weight per plant [NFW (mg)/plant], number of pods per plant (NOP/plant), number of seeds per plant (NOS/plant), seed yield [SY (g)/plant], and 100 seed weight [100 SW (g)/plant]. Because NON is the ability of the nodulated plant to initiate symbiotic associations with rhizobia, a higher nodule count generally indicates enhanced opportunities for nitrogen fixation. Furthermore, efficiency also depends on nodule activity; therefore, observations on NFW were recorded. This trait is directly associated with metabolic activity and nitrogen fixation capacity. Larger and healthier nodules indicate effective rhizobial colonization and improved nitrogen assimilation.

### Trait phenotyping for chickpea genotypes

The phenotyping for nodulation traits was done at 60 days after sowing (DAS), where plants were at 50% flowering stage. No external inoculation of *Rhizobium* was done to capture the natural variation of nodulation and associated traits. Five randomly selected plants from each genotype were uprooted with the adhered soil mass using a hand hoe by digging 20 cm or even deeper into the soil based on the plant growth and root length observed. Particular care was taken not to disturb the root nodule system during the sampling and removal of adhered soil. Root and shoot systems were separated. Roots with intact nodules were washed and counted for NON and stored in butter paper bags to further take NFW. NOP, NOS, SY, and 100 SW were taken by randomly selecting five plants for each genotype. Yield and 100 SW were measured in grams. The “corrplot” (Wei and Simko, 2021). *R package* was used to estimate Pearson’s correlation among the measured traits, while the “Factoextra” R package was used to undertake the principal component analysis (PCA) for the filtered data. The frequency distribution plots were generated using the “ggplot2” package in the R environment. Best linear unbiased predictors (BLUPs) were generated using Plant Breeding Tools Version 1.4 (PB Tools, 2014). Bartolome et al. (2014).

### Genotyping

The genotypic/SNP marker data for the 284 accessions of the association panel were obtained in the HapMap format from the database of the Centre of Excellence in Genomics and Systems Biology (CEGSB), ICRISAT, Patencheru, Telangana (https://cegresources.icrisat.org/cicerseq/; Varshney et al., 2021). Out of a total of 2,470,880 raw SNPs, 355,546 SNPs were obtained for the association panel (284) and further analyzed. The following parameters were used for SNP filtering: missing data with less than 20%, minor allele frequency (MAF) cutoff at 0.05, and an additional filter for the rate of heterozygosity of less than 10% and after calculating the threshold value of the working set of SNPs (355,546) from the Bonferroni correction as 6.85. We utilized the vcf2gwas tool (Vogt et al., 2021) for filtering SNPs.

### Marker–trait association study

Identification of marker–trait associations (MTAs) from genome-wide markers was conducted following GWAS analysis. The generated genotypic data were integrated with multi-locational phenotypic data recorded for the traits NON, NFW, NOP, NOS, SY, and 100 SW. During the phenotypic analysis of multi-location augmented trial design, the checks were considered as fixed effects while the remaining factors (location, block, and new entries/genotypes) were treated as random effects. The phenotypic data were first converted into BLUPs using statistical methods [analysis of variance (ANOVA)] performed using SAS v9.4 mixed procedure Cody, R., (2018). The population structure was assessed using neighbor-joining phylogenetic tree constructed through TASSEL software and visualized through ITOL software and PCA. For PCA, we considered the first three principal components as covariates in the Genome Association and Prediction Integrated Tool (GAPIT) in R software. The extent of linkage disequilibrium (LD) between the SNP markers was analyzed by calculating the LD (*r*^2^) values in TASSEL. The only *r*^2^ values with *p* < 0.05 within each chromosome were considered for LD decay analysis. LD decay plots were drawn by using *r*^2^ and physical distances (bp) measured by using the script following R version 4.1.1. Chromosome-wise SNP distribution with the “SNP density plot” was generated using the web tool SR-Plots (https://www.bioinformatics.com.cn/en). To identify significant MTAs, GWAS was carried out utilizing the GAPIT3 package Wang and Zhang, 2021) by employing Bayesian-information and Linkage-disequilibrium Iteratively Nested Keyway (BLINK) and fixed and random model circulating probability unification (FarmCPU) models (Liu et al., 2016). The Manhattan and quantile–quantile (Q–Q) plots were generated from qqman version 0.1.8 (Turner, 2018) using the rMVP package (0.99.17; https://github.com/xiaolei-lab/rMVP). The BLASTn search for the chickpea genome of GCA 000331145.1 on the NCBI database (https://blast.ncbi.nlm.nih.gov) was performed to investigate the genomic locations of the significant SNPs in order to find their corresponding genes.

## Results

### Dispersal and associations among nodulation and yield traits

ANOVA, frequency distribution, and correlation for the phenotypic data of the association panel collected under all the environments were statistically analyzed and the results are presented below (**Table 1**).

**Table 1:** Descriptive statistics and combined analysis of variance for the association panel.

Trait	Mean	Minimum	Maximum	Heritability (bs)	SD(d)	CV	MSS
NON	21.46	5.53	80	95.6	5.838	36.75	177.35***
NFW	204.56	141.53	4646.00	96.83	245.67	60.87	330,997.57**
N0P	34.53	7.12	123.68	91.35	6.987	26.246	671.68***
NOS	43.62	7.60	113.40	92.32	7.667	27.507	653.00***
Yield	21.32	16.07	73.71	90.33	3.89	24.180	271.01**
100 SW	22.51	7.12	123.68	92.34	4.231	22.329	282.98**

* and *** indicate 0.01 and 0.001 significance levels respectively. ** represents the level of significance and indicates a stronger significance.

**Figure 1:**
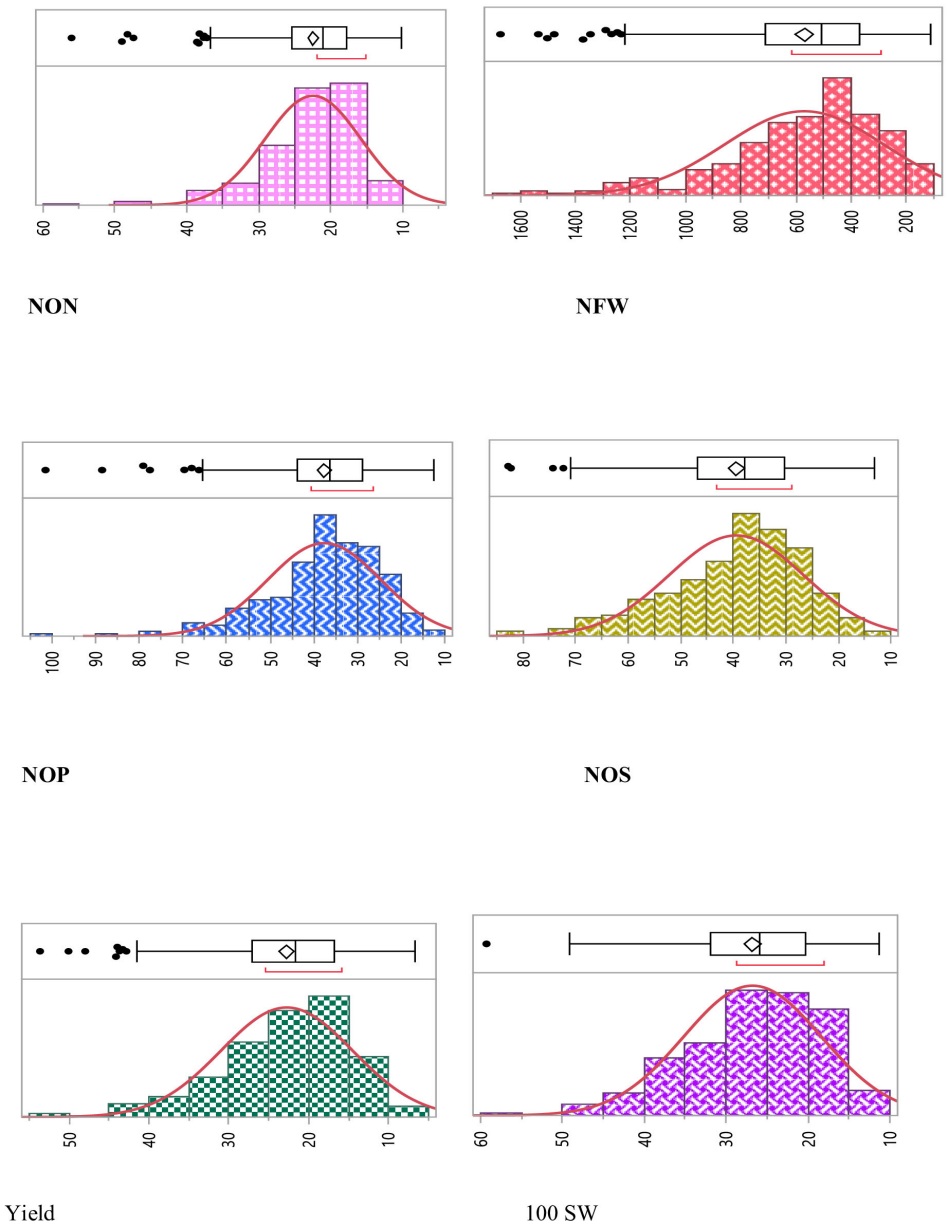
Phenotypic variation for studied traits assayed within the chickpea association panel. Within each histogram plot, the bold dashed line represents the median. The range and median for each trait are specified in the respective grid.

Thus, notable statistically significant variations were observed for all the studied traits (*p* < 0.01). Frequency distribution depicted that all the studied traits were distributed normally in the population (**Figure 1**).

**Figure 2:**
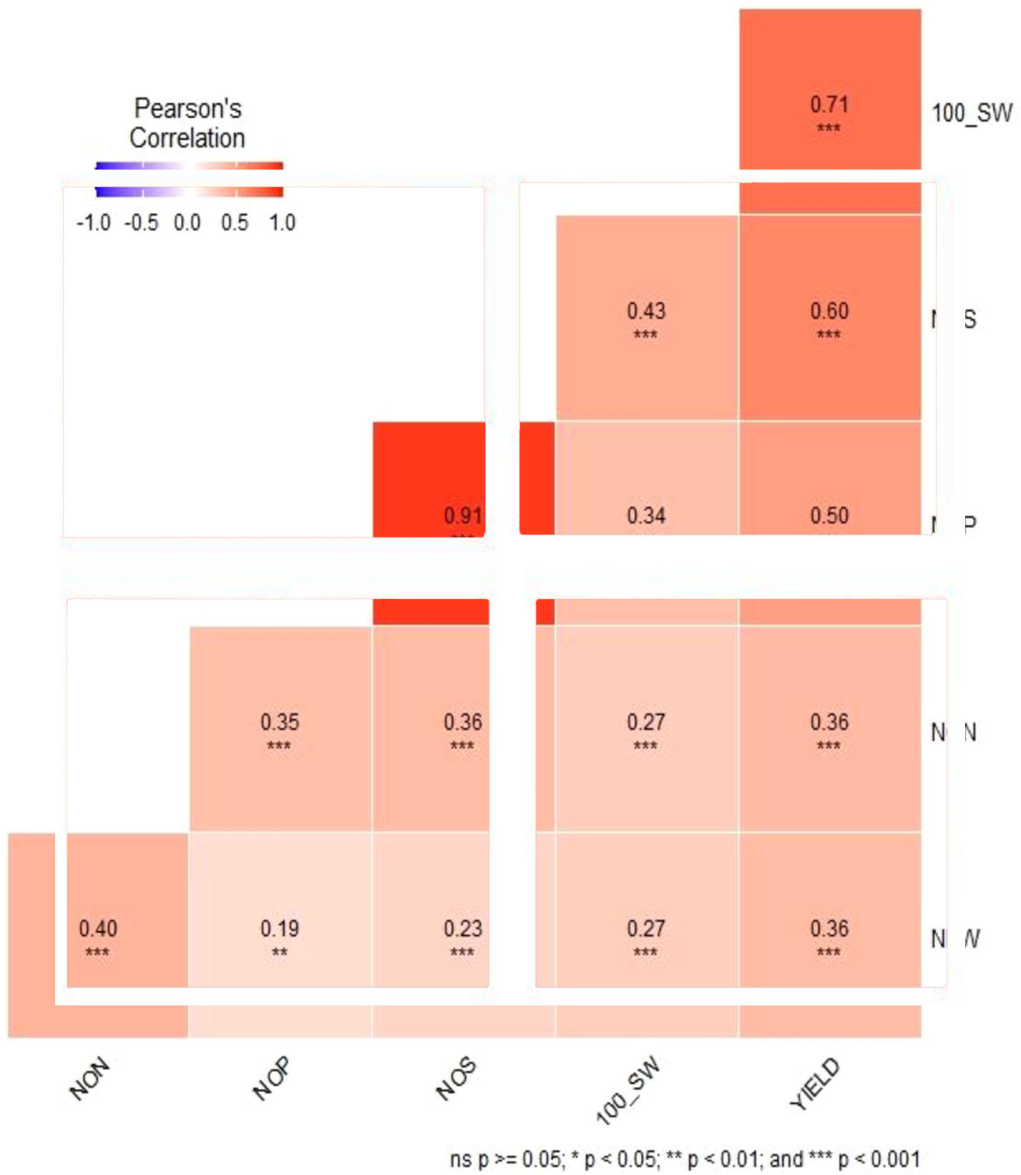
Pearson correlation analysis of six traits evaluated using the chickpea reference set. *Significant at the <0.05 level. Red indicates positive correlations, and blue indicates negative correlations among traits.

Combined descriptive statistics like minimum, maximum, mean, standard deviation, and coefficient of variation for all the studied traits for all environments were calculated. Based on the descriptive statistics, NON ranged from 5.53 to 80, NFW ranged from 141.53 to 4,646.0, NOP ranged from 7.12 to 123.68, NOS ranged from 7.60 to 113.40, SY ranged from 16.0 to 73.71, and 100 SW ranged from 7.12 to 123.68. Pearson correlation coefficient among different traits indicated that there was a significant positive correlation between NON and NFW with the yield of the studied genotypes along with positive correlation among all the studied traits (**Figure 2**).

**Figure 3:**
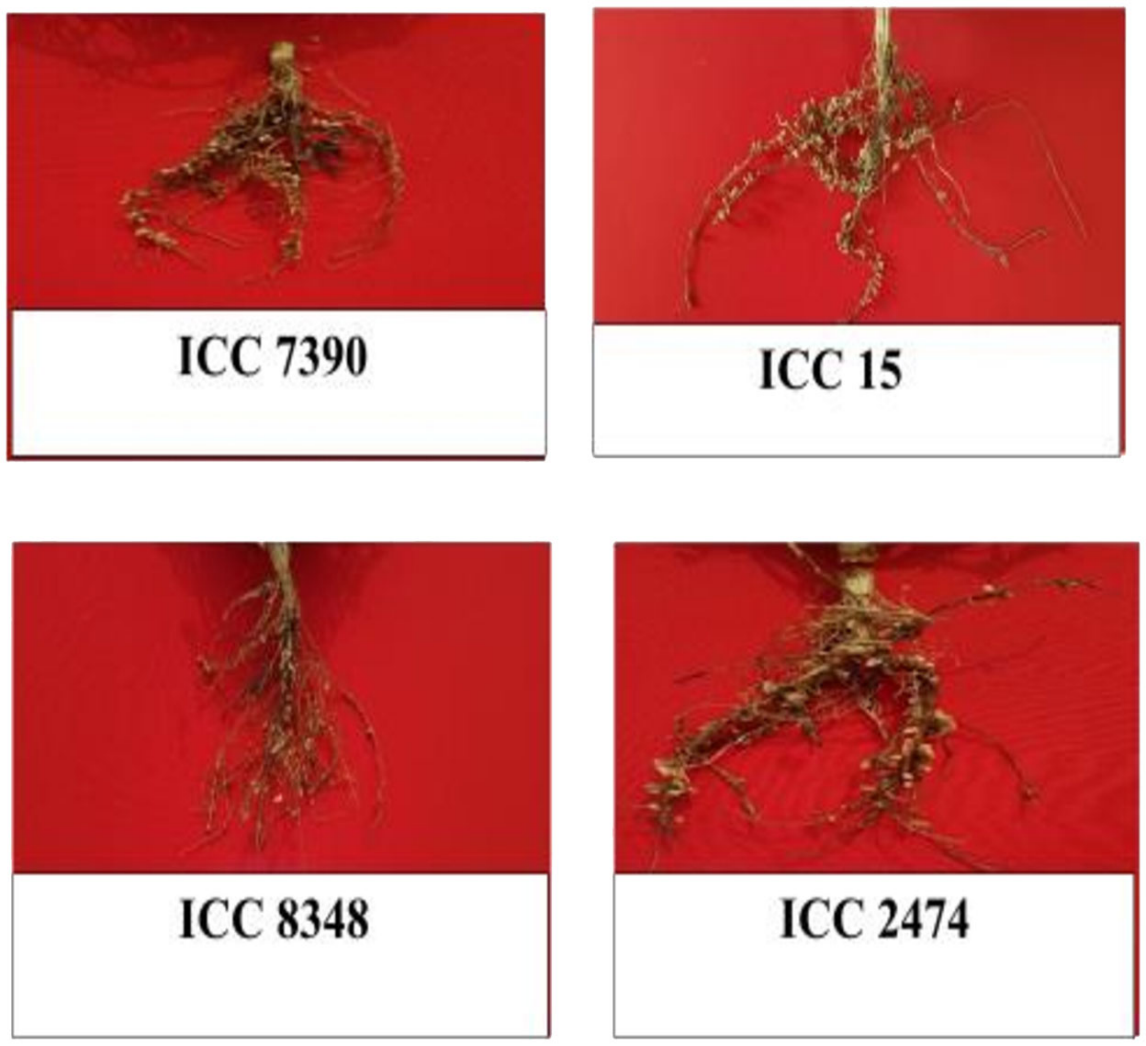
Genotypes identified as high nodulation and high yielding across the location within the chickpea association panel.

Based on mean of genotypes across the locations, the highly nodulating genotypes found in the association panel are presented (**Figure 3**). The premise was to identify a set of accessions (ICC 7390, ICC 15, ICC 8348, and ICC 2474) that can be incorporated in breeding programs for enhancing nodulation traits without adversely affecting other agronomic traits screened in different environments.

### Genotypic characteristics and population diversity analysis

**Figure 4:**
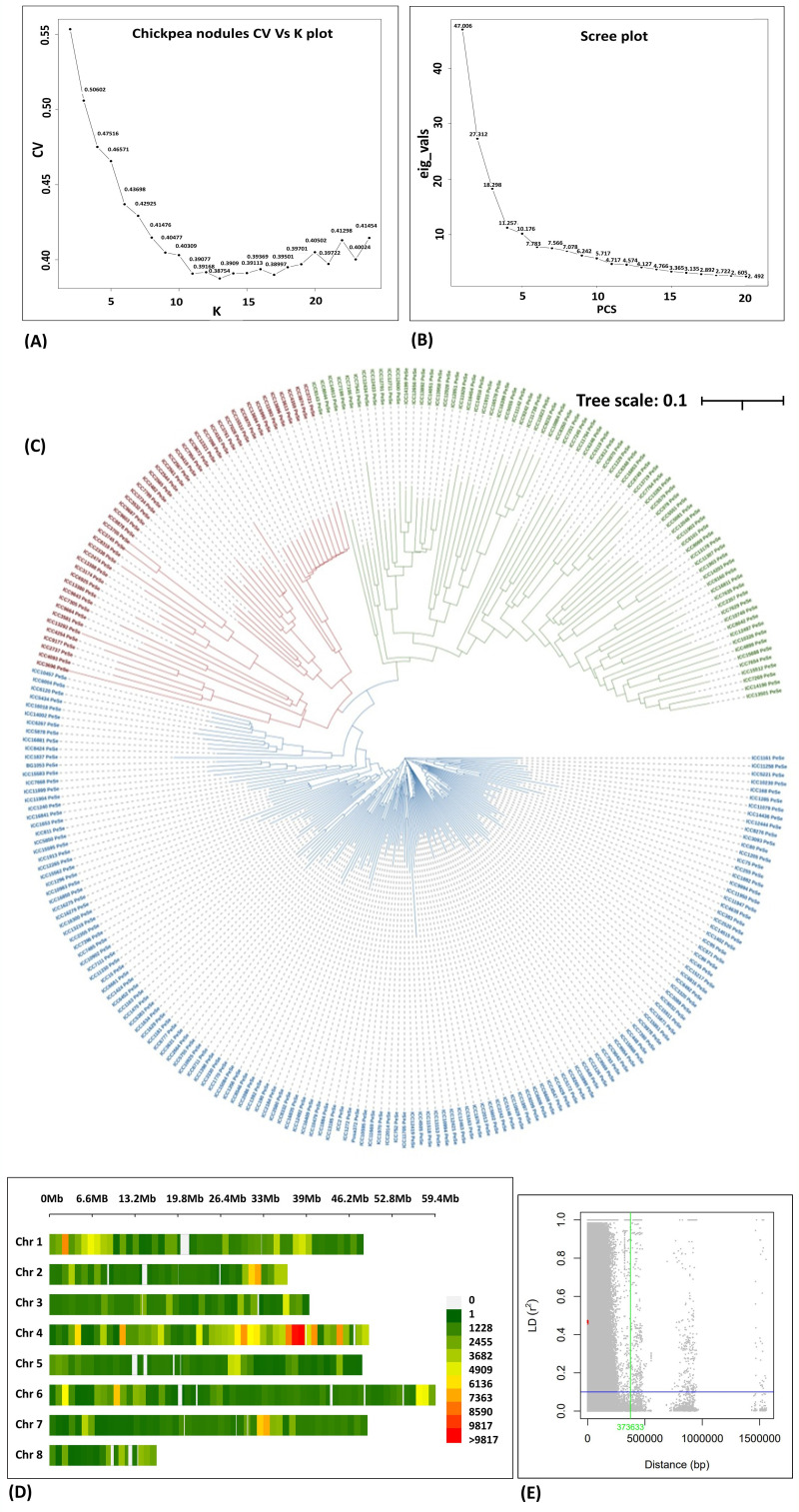
**(A)** Ancestry proportions from ADMIXTURE analysis (*k* = 3), optimal with the lowest cross-validation error. Each colored vertical line indicates the proportion of ancestral population *k*. for each accession. The numbers on the *X* axis represent the association panel accessions, and CV values are shown on the *Y* axis. **(B)** 2D plot of the principal component-based grouping is shown on the *X* axis, and eigen values are shown on the *Y* axis. **(C)** Diversity using the unweighted neighbor-joining tree method. The total number of genotypes is divided into three main clusters and represented in different colors. **(D)** SNP density plot indicating the distribution of filtered SNPs across the chromosomes in the association panel. **(E)** Linkage disequilibrium decay curve of the association panel plotting the measured *r*^2^ (*Y* axis) vs. the physical distance between pairs of SNP markers (*X* axis) (Plink 1.9).

Population structure was assessed by admixture (**Figure 4A**), PCA (**Figure 4B**), and phylogenetic tree (**Figure 4C**). The admixture plot provides valuable insights into the genetic diversity and structure of the study population. The ancestral populations (*K*) on the *X* axis vs. cross-validation on the *Y* axis were used to determine the optimal number of genetic clusters or *k* in a data set when performing admixture analysis, and we found good CV at *k* = 16. PCA revealed that the population could be divided into three groups with significant genetic variations among the genotypes studied (**Figures 4A, B**). The presence of the three sub-populations was further confirmed from the neighbor-joining diversity tree (**Figure 4C**).

### Linkage disequilibrium and LD decay

The SNP density per chromosome ranged from 1,102 to 8,802 SNPs. The LD across the genome was estimated from the HapMap file containing 355,546 SNPs, and the average LD estimated across the genome was 635.9 kb (**Figures 4D, E**, respectively). Chromosome 4 contained a greater number of SNPs and chromosome 8 had the lowest number of SNPs. In our study, a huge number of high-quality SNPs were found in GWAS that increased the probability of detecting all the possible causal variants of the traits under study.

### Marker–trait association for nodulation and yield traits

**Figure 5:**
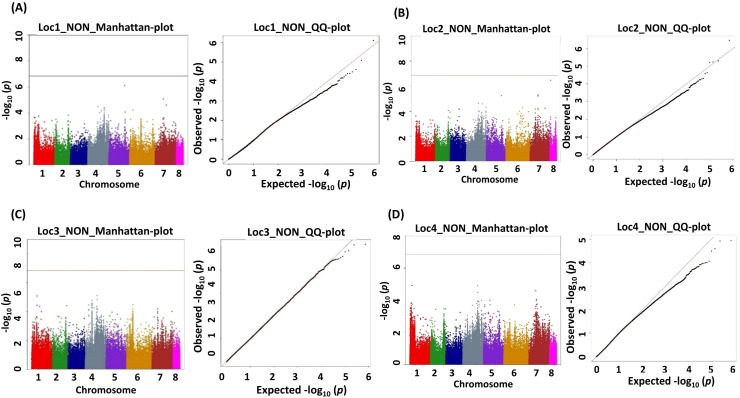
Manhattan (chromosome on the *X* axis and −log *p*-values on the *Y* axis) and quantile–quantile (Q–Q) plots of genome-wide association study (GWAS) signals illustrating SNPs linked to number of nodules (NON). **(A)** Location 1, **(B)** location 2, **(C)** location 3, and **(D)** location 4. Statistical significance threshold is shown with the green horizontal line along with their corresponding statistical significance represented by the Q–Q plot for the BLINK model.

**Figure 6:**
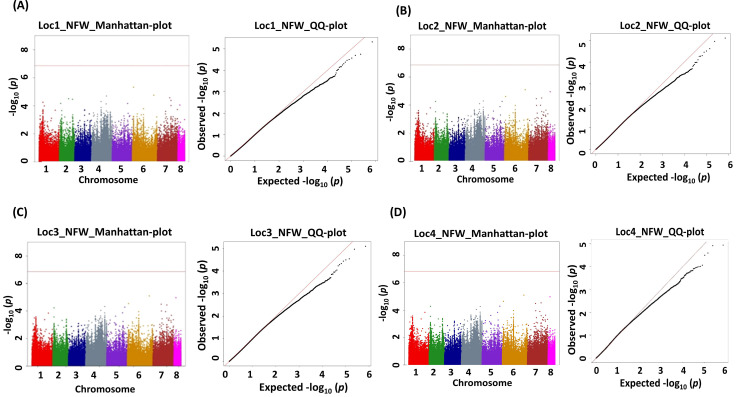
Manhattan (chromosome on the *X* axis and −log *p*-values on the Y axis) and quantile–quantile (Q–Q) plots of genome-wide association study (GWAS) signals illustrating SNPs linked to nodule fresh weight (NFW). **(A)** Location 1, **(B)** location 2, **(C)** location 3, and **(D)** location 4. Statistical significance threshold is shown with the red horizontal line along with their corresponding statistical significance represented by the Q–Q plot for the BLINK model.

A genome-wide association analysis for traits under study was conducted using BLINK and FarmCPU models. The Bonferroni correction threshold value of –log10 > 7.0 (*p*-value) was used as the cutoff, which is highly significant to identify the significant SNPs associated with the studied traits. The markers considered to be significantly associated with tested traits were represented by illustrating the Manhattan plots (**Figures 5**, **6**).

This study identified a total of 632 significantly associated SNPs by both BLINK and FarmCPU models (**Supplementary Tables 2**–**7**) and Manhattan plots (**Supplementary Figures 1**–**3**). MTA for the trait NON resulted in the identification of 27 SNPs from BLINK and 38 SNPs from FarmCPU models having 15 markers common between these two models (**Table 2**).

**Table 2:** Significant MTAs at Bonferroni-corrected *p*-values for NON in the association panel.

Location 1				
SNP	Chromosome	Position	P. value	MAF
Ca1_10074058	1	10074058	2.65E-09	0.498
Ca1_19310421	1	19310421	2.65E-09	0.498
Ca2_825897	2	825897	2.65E-09	0.498
Ca2_825900	2	825900	2.65E-09	0.498
Ca2_825902	2	825902	2.65E-09	0.498
Ca2_12931949	2	12931949	2.65E-09	0.498
Ca3_18170906	3	18170906	2.65E-09	0.498
Ca3_21635766	3	21635766	2.65E-09	0.498
Ca4_2035604	4	2035604	2.65E-09	0.498
Ca4_35572500	4	35572500	2.65E-09	0.147
Ca5_10672825	5	10672825	2.65E-09	0.498
Ca5_15877053	5	15877053	2.65E-09	0.498
Ca5_15877056	5	15877056	2.65E-09	0.498
Ca5_15877077	5	15877077	2.65E-09	0.498
Ca6_17004488	6	17004488	2.65E-09	0.498
Ca6_33340775	6	33340775	2.65E-09	0.004
Ca6_34986573	6	34986573	2.65E-09	0.498
Ca6_42172883	6	42172883	2.65E-09	0.498
Ca7_13140844	7	13140844	2.65E-09	0.498
Ca7_45149648	7	45149648	2.65E-09	0.498
Ca8_10376672	8	10376672	2.65E-09	0.498
Location 3				
Ca2_825904	2	825904	2.2E-10	0.500
Location 4				
Ca2_825897	2	825897	2.05E-12	0.498
Ca2_825900	2	825900	2.05E-12	0.498
Ca2_825902	2	825902	2.05E-12	0.498
Ca7_25313737	7	25313737	2.05E-10	0.079
Ca7_32613892	7	32613892	2.05E-12	0.498

The MTAs Ca7_32613892, Ca2_825900, and Ca2_825902 explained phenotypic variance (PVE) with 48.80%, >25%, and >25%, respectively (**Table 3**). The MTAs of NFW demonstrated 96 SNPs from the BLINK model and 3 SNPs from the FarmCPU model (**Supplementary Table 3**), and the results from the BLINK model are shown in **Table 4**, as well as Manhattan plots along with Q–Q plots (**Figures 5**, **6**).

**Table 3:** List of stable SNPs expressed at more than one environment in the association panel.

SNP	Chr.	Position	P. Value	MAF	PVE (%)	Location
NON						
Ca2_825902	2	825902	2.05E-12	0.498	27.33	1, 3and 4.
Ca2_825900	2	825900	2.08E-12	0.498	2.97	1, 3 and 4.
Ca4_2035604	4	2035604	2.08E-12	0.498	1.48	1 and 3.
Ca5_10672825	5	10672825	2.08E-12	0.498	1.11	1 and 3.
Ca5_15877053	5	15877053	2.08E-12	0.498	1.14	1 and 3.
Ca5_15877056	5	15877056	2.08E-12	0.498	2.04	1 and 3
Ca5_15877077	5	15877077	2.08E-12	0.498	1.88	1 and 3.
Ca6_33295985	6	33295985	2.08E-12	0.498	1.5	1 and 3.
Ca7_32113892	7	32113892	2.08E-12	0.498	2.05	1 and 3.
NOP						
Ca2_35665153	2	35665153	3.65E-08	0.313	1.935	2 and 3.
Ca3_36977205	3	36977205	2.26E-25	0.079	0.686	2 and 3.
Ca5_8025701	5	8025701	2.31E-11	0.498	0.98	2 and 3.
Ca7_28894406	7	28894406	2.78E-13	0.498	4.078	1, 2 and 3.
Ca7_26699787	7	26699787	2.45E-08	0.16	2.142	1, 2 and 3.
NOS						
Ca2_6640571	2	6640571	1.24E-10	0.49	7.63179	1 and 2.
Ca2_28044509	2	28044509	1.24E-10	0.27	1.572	1 and 3.
Ca2_35665153	2	35665153	4.55E-08	0.31	1.50195	1 and 3.
Ca3_16181793	3	16181793	1.24E-10	0.023	0.12635	1, 3 and 2.
Ca3_25889265	3	25889265	1.24E-10	0.023	0.55216	1 and 2.
Ca3_25890824	3	25890824	1.24E-10	0.024	1.47601	1 and 2.
Ca6_28444404	6	28444404	1.24E-10	0.494	6.05253	1, 2 and 3.
Ca7_28894406	7	28894406	1.24E-08	0.362	2.75528	1 and 2.
100 SW						
Ca1_36724701	1	36724701	9.86E-10	0.5	3.21701	1, 2 and 4.
Ca7_18587603	7	18587603	9.86E-10	0.5	2.33419	1, 2 and 4.
Ca7_18587607	7	18587607	9.86E-10	0.5	9.74799	1, 2 and 4.
Ca8_10381502	8	10381502	9.86E-10	0.5	1.67965	1, 2 and 4.
Ca1_29824861	1	29824861	2.40E-08	0	4.36E-05	1 and 4.
Ca5_10362633	5	10362633	4.30E-103	0.48	0.35649	1 and 4.
Ca5_10672825	5	10672825	4.30E-101	0.5	0.00058	1 and 4.
Ca5_15877053	5	15877053	4.30E-101	0.5	0.0002	1 and 4.
Ca5_15877056	5	15877056	4.30E-101	0.5	0.00015	1 and 4.
Ca5_15877077	5	15877077	4.30E-101	0.5	0.00051	1 and 4.
Ca6_1547764	6	1547764	3.46E-25	0.5	0.00013	1 and 4.
Ca6_1547784	6	1547784	3.46E-25	0.5	0.00017	1 and 4
Ca6_3249867	6	3249867	2.40E-08	0	0.00016	1 and 4.
Ca6_3249868	6	3249868	2.40E-08	0.5	2.55E-05	1 and 4.
Ca6_17004488	6	17004488	4.30E-101	0.5	8.33E-06	1 and 4.
Ca6_33340746	6	33340746	2.40E-08	0.5	0.00051	1 and 4.
Ca6_42172883	6	42172883	4.30E-101	0.5	0.00084	1 and 4.
Ca6_56503683	6	56503683	2.40E-08	0.5	5.67E-06	1 and 4.
Ca6_56503687	6	56503687	2.40E-08	0.5	7.03E-05	1 and 4.
Ca7_18587603	7	18587603	4.30E-103	0.5	0.03594	1 and 4.
Ca7_18587607	7	18587607	4.30E-103	0.5	0.01589	1 and 4.
Ca7_24683129	7	24683129	4.30E-103	0.08	0.06612	1 and 4.
Ca7_32613989	7	32613989	2.40E-08	0.5	3.45E-05	1 and 4.
Ca7_32613990	7	32613990	2.40E-08	0.5	5.76E-06	1 and 4.
Ca7_32613994	7	32613994	2.40E-08	0.5	0.00065	1 and 4
Ca7_32614002	7	32614002	2.40E-08	0.5	0.00145	1 and 4.
Ca7_45149648	7	45149648	4.30E-101	0.5	0.00045	1 and 4.
Ca8_9974279	8	9974279	2.40E-08	0.5	8.95E-05	1 and 4.
Ca8_10376672	8	10376672	4.30E-101	0.5	0.00103	1 and 4.
Ca8_10381502	8	10381502	4.30E-103	0.5	0.01125	1 and 4.

**Table 4:** Significant MTAs at Bonferroni-corrected *p*-values for NFW in the association panel.

SNP	Chromosome	Position	P. value	MAF
Location 1				
Ca1_10074058	1	10074058	2.18E-23	0.498168498
Ca1_13009592	1	13009592	1.28E-72	0.498168498
Ca1_18327732	1	18327732	3.18E-104	0.496336996
Ca1_19310421	1	19310421	2.18E-23	0.498168498
Ca1_21570909	1	21570909	7.52E-13	0.498168498
Ca1_22132899	1	22132899	3.51E-09	0.498168498
Ca1_22776731	1	22776731	6.59E-11	0.498168498
Ca1_25612830	1	25612830	1.12E-11	0.498168498
Ca1_28905467	1	28905467	6.14E-08	0.498168498
Ca1_29852105	1	29850105	1.12E-11	0.498168498
Ca1_36727033	1	36727033	1.12E-11	0.498168498
Ca1_37129617	1	37129617	7.52E-13	0.498168498
Ca2_825897	2	825897	3.18E-104	0.498168498
Ca2_825900	2	825900	3.18E-104	0.498168498
Ca2_825902	2	825902	3.18E-104	0.498168498
Ca2_6641754	2	6641754	7.52E-13	0.498168498
Ca2_10778915	2	10778915	3.04E-20	0.498168498
Ca2_10779028	2	10779028	3.04E-20	0.498168498
Ca2_10779156	2	10779156	3.04E-20	0.498168498
Ca2_10779159	2	10779159	3.04E-20	0.498168498
Ca2_10779270	2	10779270	3.04E-20	0.003663004
Ca2_10779338	2	10779338	3.04E-20	0.498168498
Ca2_10806147	2	10806147	1.82E-12	0.498168498
Ca2_10806174	2	10806174	1.82E-12	0.498168498
Ca2_10806191	2	10806191	1.82E-12	0.498168498
Ca2_10824354	2	10824354	3.18E-104	0.071428571
Ca2_12931949	2	12931949	2.18E-23	0.498168498
Ca2_12937693	2	12937693	3.18E-102	0.498168498
Ca2_17991269	2	17991269	1.81E-08	0.062271062
Ca2_28252608	2	28252608	7.52E-13	0.498168498
Ca2_29071303	2	29071303	7.52E-13	0.498168498
Ca2_29241002	2	29241002	3.98E-13	0.498168498
Ca2_29241003	2	29241003	3.98E-13	0.003663004
Ca2_33216048	2	33216048	3.18E-104	0.496336996
Ca2_34536551	2	34536551	3.98E-13	0.498168498
Ca3_4688867	3	4688867	3.18E-104	0.496336996
Ca3_4966928	3	4966928	3.18E-102	0.498168498
Ca3_4976598	3	4976598	3.18E-102	0.498168498
Ca3_4976629	3	4976629	3.18E-102	0.498168498
Ca3_9567318	3	9567318	3.18E-104	0.062271062
Ca3_16197582	3	16197582	7.52E-13	0.498168498
Ca3_17491433	3	17491433	6.14E-08	0.498168498
Ca3_25992547	3	25992547	6.14E-08	0.498168498
Ca3_25992548	3	25992548	6.14E-08	0.498168498
Location 3				
Ca4_7638100	4	7638100	3.18E-102	0.498168498
Ca4_25397049	4	25397049	3.51E-09	0.498168498
Ca4_33912436	4	33912436	6.92E-11	0.498168498
Ca4_33912446	4	33912446	7.52E-13	0.498168498
Ca4_33912448	4	33912448	7.52E-13	0.498168498
Ca4_33912469	4	33912469	7.52E-13	0.498168498
Ca4_35866622	4	35866622	6.92E-11	0.498168498
Ca4_43006195	4	43006195	7.52E-13	0.498168498
Ca4_43006199	4	43006199	7.52E-13	0.498168498
Ca5_6907399	5	6907399	7.52E-13	0.003663004
Ca5_6907407	5	6907407	7.52E-13	0.498168498
Ca5_6907409	5	6907409	7.52E-13	0.498168498
Ca5_10677668	5	10677668	6.48E-11	0.498168498
Ca5_10688886	5	10688886	1.12E-11	0.498168498
Ca5_21727560	5	21727560	6.59E-11	0.498168498
Ca6_1544021	6	1544021	7.52E-13	0.003663004
Ca6_1544036	6	1544036	7.52E-13	0.498168498
Ca6_15991579	6	15991579	7.52E-13	0.498168498
Ca6_28334178	6	28334178	7.52E-13	0.498168498
Ca6_28334634	6	28334634	7.52E-13	0.498168498
Ca6_33094358	6	33094358	6.92E-11	0.498168498
Ca6_33094876	6	33094876	6.14E-08	0.498168498
Ca6_33228569	6	33228569	6.92E-11	0.498168498
Ca6_33295842	6	33295842	6.14E-08	0.498168498
Ca6_33340878	6	33340878	2.17E-08	0.498168498
Ca6_33341349	6	33341349	7.52E-13	0.498168498
Ca6_37981480	6	37981480	1.52E-08	0.498168498
Ca6_50791651	6	50791651	2.17E-08	0.498168498
Ca6_50791710	6	50791710	6.59E-11	0.498168498
Ca6_56505167	6	56505167	6.21E-09	0.498168498
Ca7_909662	7	909662	3.51E-09	0.498168498
Ca7_2168538	7	2168538	7.52E-13	0.498168498
Ca7_13140844	7	13140844	2.18E-23	0.498168498
Ca7_25206921	7	25206921	7.52E-13	0.498168498
Ca7_30208029	7	30208029	7.52E-13	0.498168498
Ca7_37622722	7	37622722	1.12E-11	0.498168498
Ca8_6527794	8	6527794	6.14E-08	0.498168498
Ca8_7927537	8	7927537	6.92E-11	0.498168498
Ca8_7954617	8	7954617	6.14E-08	0.498168498
Ca8_8074306	8	8074306	1.52E-08	0.498168498
Ca8_10590208	8	10590208	2.17E-08	0.498168498
Ca8_10887378	8	10887378	2.95E-09	0.498168498
Ca8_13815453	8	13815453	7.52E-13	0.003663004
Ca8_13815469	8	13815469	7.52E-13	0.498168498

The MTAs Ca1_28905467 and Ca1_29852105 explained 3.2% and 9.2% of PVE for respective SNPs of NFW. The MTAs of NOP recognized 43 SNPs from BLINK and 60 SNPs from FarmCPU, respectively, having 17 SNPs common with the BLINK model. MTAs of NOS identified 16 and 59 SNPs from the BLINK and FarmCPU models, respectively, with 12 SNPs being common. Furthermore, for the trait 100 SW, we have found 4 and 286 MTAs from the BLINK and farm CPU models, respectively. Thus, the maximum number of SNPs was identified for 100 SW followed by NFW, NON, and NOP.

### Marker–trait associations expressed consistently across the environments

The SNPs found for more than two locations were considered as stable SNPs in our study (**Table 3**). The two SNPs Ca1_36724701 and Ca7_18587603 present on chromosomes 1 and 7, respectively, belonging to the trait NON were found to be stable at locations 1, 3, and 4. Seven SNPs were found to be stable at locations 1 and 3 for NON. The stable SNP 2_825902, which is found on chromosome 2, explained 27.33% of the PVE. Furthermore, 7 stable SNPs for NFW, 5 for NOP, 6 for NOS, and 32 for 100 SW were identified. The PVE explained by the stable MTAs ranged from 1.11% to 27.33% for NON, 5.7% to 34.46% for NFW, 1.12% to 8.95% for NOP, 1.23% to 11.71% for NOS, 1.23% to 31.45% for seed yield, and 0.92% to 28.65% for 100 SW. Variation in PVE % was observed across different locations. This may be attributed to the genotype-by-environment (G×E) interactions, which influence the relative performance of the genotypes under different environmental conditions.

### Associated SNPs with two or more traits

The SNPs found common for two or more traits were considered as pleotropic (**Table 5**). The three SNPs (Ca1_10074058, Ca2_825900, and Ca2_825902) were found to be common for the traits NON and NFW. The SNP Ca7_18587607 located on chromosome number 7 was found to be common for the traits NOP, NOS, and 100 SW. We also found 11 significant and common SNPs for the traits NOP and 100 SW. Significant SNPs contribute substantially to the variability of the trait in the population being studied. Researchers prioritize these SNPs for further investigation and consider them as potential candidates for explaining the genetic basis of the trait.

**Table 5:** List of the common MTAs identified for more than one trait.

S.No	SNP	Chromosome	Position	P. value	Trait
1	Ca1_10074058	1	10074058	2.18E-13	NON and NFW.
2	Ca2_825900	2	825900	3.18E-15	NON and NFW.
3	Ca2_825902	2	825902	3.18E-15	NON and NFW.
4	Ca6_28444404	6	28444404	2.26E-25	NOP and NOS.
5	Ca7_18587607	7	18587607	2.26E-25	NOPNOS and 100 SW.
6	Ca7_26699787	7	26699787	2.85E-15	NOP and NOS.
7	Ca7_28894406	7	28894406	2.03E-17	NOP and NOS.
8	Ca7_18587603	7	18587603	4.28E-12	NOP, NOS and 100 SW.
9	Ca3_25885767	3	25885767	4.28E-12	NOS and 100 SW.
10	Ca3_25889265	3	25889265	4.28E-12	NOS and 100 SW.
11	Ca3_25890824	3	25890824	4.28E-12	NOS and100 SW.
12	Ca6_28444404	6	28444404	4.28E-12	NOS and100 SW.
13	Ca1_36724701	1	36724701	4.28E-12	NOP and 100 SW.
14	Ca3_36977205	3	36977205	4.28E-10	NOP and 100 SW.
15	Ca5_711446	5	711446	1.77E-10	NOP and 100 SW.
16	Ca6_1492142	6	1492142	1.77E-12	NOP and 100 SW.
17	Ca7_18587603	7	18587603	4.28E-10	NOP and 100 SW.
18	Ca7_18587607	7	18587607	4.28E-10	NOP and 100 SW.
19	Ca7_24683129	7	24683129	4.28E-10	NOP and 100 SW.
20	Ca8_7955493	8	7955493	1.77E-12	NOP and 100SW.
21	Ca8_10381502	8	10381502	4.28E-10	NOP and 100 SW.

### Candidate genes and associated SNPs

The significantly associated SNPs with different traits were further used for the identification of putative candidate genes based on the position of SNPs and flanking regions (Kawahara et al., 2013). The 10-kb upstream and 10-kb downstream sequences from the SNP positions were retrieved from the NCBI database and functionally annotated based on CDC Frontier v1 functional annotations (Varshney et al., 2013). The SnpEff-4.3T open-source program was also used for variant annotation and prediction of significant SNP effects, and we found that majority of the SNPs in our study were present as intron variants. The Ca7_32113892 identified for NON was present within the genomic regions of the protein encoding a calumenin-like isoform X2 and a calumenin B-like isoform X1. These have a function in NOD factor export and, in turn, are shown to be involved in nodulation. The SNP located on Ca7_45149648 localized with the protein transcription factor GTE4-like protein that functions as an activator of gene expression upon infection with *Pseudomonas syringae* and helps in the upregulation of salicylic acid (SA)-mediated immune defense genes was associated with NON. The GTE4-like protein is closely associated with bacterial infection and may be involved in the regulation of the nodulation process. Some of the important proteins along with their molecular functions are listed in **Table 6**.

**Table 6:** Candidate genes identified at the 10-kb region of linked SNPs along with their molecular functions for NON and NFW in the association panel.

SNP	Protein	Function
NON		
Ca7_32113892	Calumenin like isoform X2 and Calumenin B-like isoform X1	Plant growth and development and also acts as a nod factor export binding protein and light signaling.
Ca7_40986331	FY isoform X1	Flowering time control.
Ca7_45149648	Transcription factor GTE4-like	GTE4 mainly functions as activator of gene expression upon infection with *Pseudomonas syringe*, it helps in upregulation of salicylic acid (SA) mediated immune defence genes.
Ca7-28688203	Obtusifoliol 14-alpha demethylase	Involved in phytosterol synthesis and affects pollen and seed development.
Ca5_10672825	Vacuolic protein sorting-associated protein 55 homolog isoform X3	Retrograde transport of acid hydrolase receptors.
Ca5-25662323	Beta-galactosidase	Helps in germination and seedling growth
Ca5_15877053	Gibberellin 2-beta-dioxygenase 8-like	Involved in osmotic stress tolerance.
NFW		
Ca3_21635766	PREDICTED: uncharacterized protein	Oxidation-reduction process
Ca7_2168538	Mitochondrial outer membrane protein porin 4	Response to bacterium, Inorganic anion transport, anion transmembrane transport
Ca7_30208029	Tim17/Tim22/Tim23/Pmp24 family protein	Protein import into mitochondrial inner membrane.

## Discussion

The identification of chickpea germplasm with good nodulation parameters is key for developing cultivars for different end users and identifying high nodulation donors to help improve the BNF. The chickpea core collection also shows a considerable high amount of variation for nodulation traits among the studied accessions. It is believed that such genotypes exhibiting optimal nodulation play an important role in improving BNF. Phenotypic data analysis has shown that nodulation traits such as NON and NFW were positively correlated with yield per plant. Similar results were also reported by Istanbuli et al. (2024). The traits NOP and NOS were positively correlated with chickpea yield as reported earlier in several studies (Aarif et al., 2014; Dawane et al., 2020). In this study, we have identified chickpea accessions (ICC 7390, ICC 15, ICC8348, and ICC2474) across multiple locations that could be exploited as a base in breeding, especially for nodulation-associated traits. Genomics-assisted crop improvement of chickpea is usually hindered by its narrow genetic base and low intra-specific polymorphism among cultivated desi and kabuli accessions. To overcome this, large-scale discovery and genotyping of informative sequence-based markers such as SNPs differentiating the maximum number of desi and kabuli accessions and exhibiting high intra-specific potential among cultivated accessions using a user-friendly, rapid, and cost-effective genotyping assay at the genome-wide scale is essential (Bajaj et al., 2015).

Population structure analysis provides insight into genetic variation in chickpeas that has evolved through evolutionary processes such as genetic drift, demographic history, and natural selection (Andam et al., 2017). In our study population, stratification has been with three clusters/sub-populations independent of biological status and seed type. In accordance with the results obtained in this study, a study by Varshney et al. (2019) also reported the presence of three sub-populations using genome-wide SNP markers. In another study, four sub-populations were revealed in a diverse set consisting of 186 chickpea genotypes, using DArT-seq markers (Farahani et al., 2019). The presence of three sub-population aligns with a previous study on desi and kabuli chickpea genotypes, which emphasizes the importance of geographic origin and adaptive environments in genotype clustering (Basu et al., 2018). Further investigation revealed that most sub-populations were admixed, which could be useful in breeding programs to produce hybrids with desirable traits. These findings are supported by the pure and minimally admixed accessions studied by Bajaj et al. (2015). GWAS overcomes two common limitations of the traditional linkage mapping, viz., restricted allelic diversity and limited genetic resolution (Brachi et al., 2011). Owing to its high resolution, cost-effectiveness, and non-essential pedigrees, association mapping has been able to dissect many important traits in chickpea, such as concentration of mineral nutrients (Diapari et al., 2014; Upadhyaya et al., 2016; Fayaz et al., 2022; Samineni et al., 2022); seed yield (Basu et al., 2018); drought tolerance (Li et al., 2018); root morphology, phosphorous acquisition, and use efficiency (Thudi et al., 2021); and salinity tolerance (Ahmed et al., 2021).

In recent years, GWAS has become crucial for pinpointing SNPs in chickpeas and helping us identify genomic variations linked to specific traits (Roorkiwal et al., 2022). GWAS have been performed for location and trait-wise individual genotypes for nodulation and yield contributing traits. The present study identified SNPs that are common across two or more traits, which may indicate pleiotropy. However, majority of them controlled the related traits such 3 SNPs common for NON and NFW, 4 SNPs common for NOS and NOP, and 14 SNPs common for NOP and 100 SW, and some SNPs were found common for NOP, NOS, and 100 SW. Also, GWAS analysis of the association panel led to the identification of stable SNPs with good PVE values for all the traits under study as presented in the Results section. For the trait NON, SNP Ca2_825900 was found to be stable with 27.32% of PVE and the SNP Ca7_32613892 was found to explain 48.11% of PVE. We also found that most of the SNPs common across the models indicate the true association of markers with the trait of interest. SNP Ca1_19310421 associated with the NON trait is situated in a genomic region where the gene codes for an SNF1-related protein kinase regulatory subunit beta-3. This subunit is implicated in the regulation of protein kinase activity, cellular responses to nitrogen levels, and the intricate response to sucrose signaling (Jamsheer et al., 2022). These findings collectively contribute to our understanding of the genetic underpinnings of the observed traits and the multifaceted processes governing plant nutrient interactions and developmental response. MTAs identified for the NFW also had prominent candidate genes: Ca1_29852482 is located on chromosome number 1 that involves genes for the regulation of plant immunity (Ashraf et al., 2018), the SNP Ca1_28905467 located on chromosome 1 codes for environmental stress (Che et al., 2020), and the SNP Ca8_12988095 is located on the nearest gene that codes for plant–microbe interactions (Liu et al., 2023). The presence of nodulation-specific and other related candidate genes supports earlier findings on the discovery of SNPs Ca1pos289.52482.1 and 6_33340878 (Chandana et al., 2024).

Thus, this study may be considered as a complementary work of our previous accomplishments with a new set of association panel that also validates already identified markers for nodulation. The validated SNPs Ca1pos289.52482.1 and 6_33340878 can be converted to Kompetitive allele-specific PCR (KASP) markers and utilized in the marker-assisted breeding for genes related to nodulation and dissecting the BNF along with yield and productivity. Based on our study, we found that the genomic regions controlling NON are expected to be present on chromosome number 7 with a size of 286 to 451 mb. The MTAs have revealed that several genomic regions on chromosome number 5, with lengths ranging from 158 to 217 Mb, contain SNPs that contribute to the NON trait. The genomic regions controlling the nodules’ fresh weight are present on chromosome 6. The BNF is a sustainable and globally applicable means to supply nitrogen to agricultural systems. An effective strategy to augment BNF involves the breeding and utilization of legume genotypes possessing enhanced BNF capacity. The germplasm lines exhibited significant trait variations encompassing all traits under study. Correlation analysis revealed compelling insights, highlighting a significant positive correlation and the direct effect of nodulation traits on yield within the chickpea population. Based on nodulation and yield-related traits, the promising accessions identified in this study can serve as potential donors for designing nodulation-rich chickpea varieties for the future. Leveraging association studies, we successfully identified noteworthy and stable MTAs linked to the nodulation traits. Phylogenetic tree and genotypic PCA confirmed three sub-populations in the association panel. LD decay estimation revealed an average LD block size of 636.8 kb, which helped us find good MTAs in our study. Highly significant markers were reported for nodulation and yield traits in chickpea. Subsequent *in silico* analysis revealed that a substantial proportion of these MTAs were situated within intergenic regions with the potential to modulate genes associated with the focal traits.

## Data Availability

The original contributions presented in the study are included in the article/**Supplementary Material**. Any further inquiries can be directed to corresponding authors.
